# Supplementary material for: A time-series analysis of blood-based biomarkers within a 25-year longitudinal dolphin cohort
Source: PLoS Comput Biol. 2023 Feb 21;19(2):e1010890. doi: 10.1371/journal.pcbi.1010890 (PMC9983899; doi:10.1371/journal.pcbi.1010890)
Supplement: S1 Text — (PDF) [file pcbi.1010890.s001.pdf]

1 Introduction

This is supplemental information for *'A time-series analysis of blood-based biomarkers within a 25-year longitudinal dolphin cohort'*. In sections 2 and 3 we describe the methods used in the main text to model the longitudinal data. In section 4 we describe the methods used to cluster the array of type-A interactions. These methods are implemented in Matlab within the github repository '`https://github.com/adirangan/dir_PAD`' referenced in the main text. In section 5 we describe our calculation of the holm-bonferroni corrected p-values. in section 6 we describe how we estimate the aging rate of the dolphins.

2 Model Structure

As described in the main text, our strategy will be to model the evolution of any d specific variables via a simple linear stochastic-differential-equation (SDE). Due to constraints we'll discuss below, we typically limit ourselves to $d = 2$, considering pairs of variables at a time. With this model the evolution over a time-interval $[t, t']$ can be approximated as:

$$dX(t) = [\boldsymbol{a} + A \cdot X(t)]\, dt + B \cdot dW(t). \tag{1}$$

In Eq 1 the vector $X(t) \in \mathbb{R}^d$ represents the d-dimensional vector-valued solution-trajectory at the initial time t, the time-increment $dt = t' - t$ represents the difference between the initial time t and the final time t', and $dX(t) = X(t') - X(t) \in \mathbb{R}^d$ represents the vector of variable-increments between times t and t'. The vector $\boldsymbol{a} \in \mathbb{R}^d$ corresponds to a constant 'velocity' for each of the variables. The matrix $A \in \mathbb{R}^{d \times d}$ represents the deterministic type-A (linear) interactions between variables. The type-B variation is modeled by the Brownian-increments $dW(t)$, each drawn independently from the Gaussian distribution $\mathcal{N}(0, dt \cdot I_{d \times d})$. The symmetric matrix $B \in \mathbb{R}^{d \times d}$ controls the anisotropy of the type-B variation; the covariance of this stochastic term is given by the symmetric-matrix $dtBB^{\intercal}$ [1]. We assume that we do not measure $X(t)$ directly, but rather some $Y(t)$ which depends on $X(t)$, and which also incorporates the type-C observation-noise:

$$Y(t) = X(t) + C \cdot \boldsymbol{\epsilon}(t), \tag{2}$$

where each vector $\boldsymbol{\epsilon}(t) \in \mathbb{R}^d$ at each observed-time is drawn (independently) from the Gaussian distribution $\mathcal{N}(0, I_{d \times d})$. The symmetric matrix $C \in \mathbb{R}^{d \times d}$ controls the anisotropy of the type-C errors; the covariance of this observation-error is given by the symmetric-matrix $CC^{\intercal}$.

Note that the observed-times are not necessarily unique: t' could very well equal t, and dt may equal 0. In this situation $dX(t) \equiv \mathbf{0}$, and so $X(t')$ will equal $X(t)$. However, because of the type-C errors, $Y(t')$ will in general be different from $Y(t)$.

Below we describe the methods we use to fit longitudinal data with the simple dynamical system (SDE) above. After fitting the model parameters we interpret A and $BB^{\intercal}$ as having potential biological significance.

3 Implementation

We'll begin by rewriting Eq 1 slightly. As written above, the velocity $\boldsymbol{a}$ will contribute a time-dependent term of the form $\boldsymbol{a}dt$ to the evolution of $X(t)$. Without loss of generality, we can account for the velocity $\boldsymbol{a}$ by subtracting a polynomial $Q(t)$ from $X(t)$:

$$Q(t) = \sum_{q=0}^{q=q_{\max}} \boldsymbol{\alpha}_q t^q \tag{3}$$

$$R(t) = X(t) - Q(t), \tag{4}$$

$$\tag{5}$$

where the $\boldsymbol{\alpha}_q \in \mathbb{R}^d$ are vector-valued coefficients of the degree $q_{\max}$ vector-valued polynomial $Q(t)$. We then assume the term $R(t)$ evolves according to:

$$dR(t) = A \cdot R(t)dt + B \cdot dW(t), \tag{6}$$

Note that the time-derivative of $Q(t)$ is given by:

$$\frac{dQ}{dt} = \sum_{q=1}^{q_{\max}} \boldsymbol{\alpha}_q q t^{q-1} = \sum_{q=0}^{q_{\max}-1} (q+1)\boldsymbol{\alpha}_{q+1} t^q,$$

allowing the original representation in Eq 1 to be represented as a special case of Eq 6 simply by setting $q_{\max} = 0$ and $\boldsymbol{a} := -A \cdot \boldsymbol{\alpha}_0$. Below we'll use the term α to refer to the collection of $\boldsymbol{\alpha}_q$; i.e., α can be thought of as an array in $\mathbb{R}^{d\times(q_{\max}+1)}$.

We'll also introduce some extra notation to track the different times at which the system is measured, along with their multiplicities. More specifically, we'll assume that the data itself involves the $j_{\max}$ observed-times $\{\tau_1, \ldots, \tau_{j_{\max}}\}$, as well as a vector of observations Y_j at each of those times. Because multiple observations might correspond to the same time (e.g., τ_j might equal τ_{j+1}), the number $k_{\max}$ of unique time-points might be smaller than $j_{\max}$. To denote these $k_{\max}$ unique time-points, we'll use the notation $\{t_1, \ldots, t_{k_{\max}}\}$. For example, below we'll denote the R-increments over a time-step via:

$$Z_k = R(t_{k+1}) - R(t_k) - A \cdot R(t_k) \cdot dt_k \quad \text{with:} \quad dt_k = t_{k+1} - t_k. \tag{7}$$

Later on we'll cross-reference the two arrays for t and τ, referring to $k(j)$ as the time-index k for which $t_k = \tau_j$.

Before discussing how we fit this model, we remark that we aren't guaranteed to measure all d components of Y_j at each observed-time τ_j; some components of Y_j may be 'missing'. These missing entries can be treated naturally, and we'll address this further below in section 3.2.

The various parameters in the model that we'll consider include α, A, B, and C. The model also involves the hidden (or 'latent') trajectory $X(t_k)$, corresponding to the 'true' (but unknown) system-state at times t_k. The values of α and A are functionally related to the hidden trajectories $Q(t_k)$, $R(t_k)$ and $X(t_k)$ as described in Eq 6. Finally, the hidden trajectory $X(t_{k(j)})$ is related to the observed measurements $Y(\tau_j)$ via Eq 2.

These quantities can be related within a standard bayesian framework:

$$P\left(Y|\alpha, A, B, C\right) P\left(\alpha, A, B, C\right) = P\left(\alpha, A, B, C|Y\right) P\left(Y\right), \tag{8}$$

with the likelihood $P\left(\alpha, A, B, C|Y\right)$ defined via:

$$P\left(\alpha, A, B, C|Y\right) = \int_X P\left(\alpha, A, B, C, X|Y\right) = \int_X P\left(\alpha, A, B, C|X\right) P\left(X|Y\right), \tag{9}$$

corresponding to an integral over all possible hidden trajectories $X(t)$.

If we were to assume a uniform prior for the model parameters, then $P\left(\alpha, A, B, C\right)$ would be a constant and we would have the familar expression:

$$P\left(Y|\alpha, A, B, C\right) \propto P\left(\alpha, A, B, C|Y\right) = \int_X P\left(\alpha, A, B, C|X\right) P\left(X|Y\right), \tag{10}$$

i.e., the probability of the data given the model is proportional to the probability of the model given the data (after marginalizing with respect to the hidden trajectory $X(t)$). Thus, to maximize the likelihood of the model we need to find parameters which maximize the likelihood $P\left(\alpha, A, B, C|Y\right)$.

In general, maximizing the likelihood in Eq 10 is challenging, as it is a non-convex function of the model parameters. To approximate a maximum-likelihood solution, we'll use an iterative-refinement which can be viewed as a version of expectation-maximization [2].

The two main formulae we'll use in our method are:

$$P\left(\alpha, A, B, C|X\right) = \prod_{k=1}^{k_{\max}-1} \frac{1}{(2\pi)^{d/2}} \frac{dt_k^{d/2}}{\sqrt{\det(BB^\intercal)}} \exp\left(-\frac{1}{2} \cdot {Z_k}^\intercal \cdot \text{inv}(BB^\intercal) \cdot Z_k\right), \tag{11}$$

$$P\left(Y|X\right) = \prod_{j=1}^{j_{\max}} \frac{1}{(2\pi)^{d/2}} \frac{1}{\sqrt{\det(CC^\intercal)}} \exp\left(-\frac{1}{2} \cdot \left[Y_j - X(t_{k(j)})\right]^\intercal \cdot \text{inv}(CC^\intercal) \cdot \left[Y_j - X(t_{k(j)})\right]\right), \tag{12}$$

with $dt_k = t_{k+1} - t_k$ and Z_k defined in Eq 7 and $k(j)$ corresponding to the t_k that equals τ_j.

By combining Eqs 11 and 12 and using bayes-rule (with a uniform prior on the hidden trajectory X), we can approximate the likelihood in Eq 10 via:

$$P\left(\alpha, A, B, C|Y\right) \propto \int_X P\left(\alpha, A, B, C|X\right) P\left(X|Y\right) \tag{13}$$

$$\propto \int_X P\left(\alpha, A, B, C|X\right) P\left(Y|X\right). \tag{14}$$

The logarithm of the integrand on the right-hand-side of Eq 14 is a quadratic function of the hidden trajectory $X(t_k)$. Consequently, the integral over X in Eq 14 corresponds to a standard Gaussian integral, and can be calculated easily for

any fixed values of α, A, B and C.

Generally speaking, we approximate the model parameters α, A, B and C iteratively. Referring to the iteration index as μ, our algorithm proceeds as follows:

initialization: We set $\mu := 0$, and initialize the array $\alpha^{[\mu]}$ and matrix $A^{[\mu]}$ to be zero and the matrices $B^{[\mu]}$ and $C^{[\mu]}$ to be the identity.

update model parameters: We use nelder-meade optimization to update the model parameters $\alpha^{[\mu]}$, $A^{[\mu]}$, $B^{[\mu]}$ and $C^{[\mu]}$ to maximize the likelihood in Eq 14. Along the way we track the maximum likelihood estimate for the hidden trajectory $X^{[\mu]}$.

termination: We stop when the likelihood converges, with a relative-error specified by a global tolerance (e.g., less than 10^{-6}).

3.1 Additional Details

Our implementation also includes the following modifications which help to accelerate convergence:

preliminary optimization: After initialization, but before beginning nelder-meade optimization, we perform several preliminary rounds of 'sequential' optimization. As an example, C can be updated by fixing the other model parameters $\alpha^{[\mu]}$, $A^{[\mu]}$ and $B^{[\mu]}$, assuming that $X^{[\mu]}$ is fixed at its (current) maximum-likelihood estimate, and then setting $C^{[\mu+1]}$ to be the value of C that maximizes the likelihood in Eq 14. We use this strategy for each of the model parameters, updating C, B, α and A in sequence. We perform this sequential optimization until the likelihood converges (which typically takes 3-4 rounds of updates when $d = 2$).

regularization: When performing the optimization mentioned above, we subtract regularization terms from the log-likelihood. These regularization terms penalize the magnitude and eccentricity of $\text{inv}(BB^\intercal)$ and $\text{inv}(CC^\intercal)$, and can be thought of as non-uniform priors for B and C. The regularization term $\mathcal{L}$ for B is:

$$\mathcal{L}(B) := (k_{\max} - 1) \cdot \frac{1}{2} \sum_{d'=1}^{d'=d} \sum_{d''=d'+1}^{d''=d} (l_{d'} - l_{d''})^2 + (k_{\max} - 1) \cdot \frac{1}{2} \sum_{d'=1}^{d'=d} l_{d'}^2,$$

where $\{l_1, \ldots, l_d\}$ are the d log-eigenvalues of $\text{inv}(BB^\intercal)$. The regularization term for C is similar:

$$\mathcal{L}(C) := j_{\max} \cdot \frac{1}{2} \sum_{d'=1}^{d'=d} \sum_{d''=d'+1}^{d''=d} (l_{d'} - l_{d''})^2 + j_{\max} \cdot \frac{1}{2} \sum_{d'=1}^{d'=d} l_{d'}^2,$$

with the l-terms now corresponding to log-eigenvalues of $\text{inv}(CC^\intercal)$.

regression: As an alternative to the explicit regularization above, one can estimate $CC^\intercal$ using the empirical covariance of the vectors $\left[Y_j - X_{k(j)}\right]$. Similarly, one can estimate $BB^\intercal$ by applying linear regression (with dependent-variable dt_k) to the empirical covariances of the Z_k. In our experience this strategy often produces results which are quite similar to the explicit regularization described above.

3.2 Dealing with missing measurements

As alluded to above, there are observed-times τ_j for which only some of the components of Y_j are observed. When calculating the likelihood for these times we average (i.e., marginalize) over the possible values for the missing entries.

As an example, consider a particular τ_j, corresponding to the time $t_{k(j)}$. The associated likelihood for this term in Eq 12 was originally:

$$P\left(Y_j | X(t_{k(j)})\right) = \frac{1}{(2\pi)^{d/2}} \frac{1}{\sqrt{\det(CC^\intercal)}} \exp\left(-\frac{1}{2} \cdot \left[Y_j - X(t_{k(j)})\right]^\intercal \cdot \mathrm{inv}(CC^\intercal) \cdot \left[Y_j - X(t_{k(j)})\right]\right),$$

which, after defining $X := X(t_{k(j)})$ and dropping the index j for readability, looks like:

$$P(Y|X) = \frac{1}{(2\pi)^{d/2}} \frac{1}{\sqrt{\det(CC^\intercal)}} \exp\left(-\frac{1}{2} \cdot [Y - X]^\intercal \cdot \mathrm{inv}(CC^\intercal) \cdot [Y - X]\right).$$

Now let's assume that only the first $d' < d$ components of Y are observed. We can write $Y \in \mathbb{R}^d$ as the concatenation of $Y^{\text{top}} \in \mathbb{R}^{d'}$ (which is known) and $Y^{\text{bot}} \in \mathbb{R}^{d-d'}$ (which is missing):

$$Y = \begin{bmatrix} Y^{\text{top}} \\ Y^{\text{bot}} \end{bmatrix},$$

doing the same for X. With this notation, the marginalized version of $P(Y|X)$ for this observed-time becomes:

$$P\left(Y^{\text{top}} \middle| \begin{bmatrix} X^{\text{top}} \\ X^{\text{bot}} \end{bmatrix}\right) = \int_{Y^{\text{bot}}} \frac{1}{(2\pi)^{d/2}} \frac{1}{\sqrt{\det(CC^\intercal)}} \exp\left(-\frac{1}{2} \cdot \begin{bmatrix} Y^{\text{top}} - X^{\text{top}} \\ Y^{\text{bot}} - X^{\text{bot}} \end{bmatrix}^\intercal \cdot \mathrm{inv}(CC^\intercal) \cdot \begin{bmatrix} Y^{\text{top}} - X^{\text{top}} \\ Y^{\text{bot}} - X^{\text{bot}} \end{bmatrix}\right) \quad (15)$$

The expression in Eq 15 is a standard Gaussian integral which can be calculated easily when C is fixed. When treating multiple missing values for Y_j across different observed-times τ_j, we simply replace each of the corresponding terms in Eq 12 with an appropriately marginalized version analogous to Eq 15.

3.3 Identifiability

Fitting the model above to a particular data-set involves maximizing the likelihood shown in Eq 14. Because this is a nonconvex optimization problem, our strategy is not guaranteed to succeed. Generically, nonconvex optimization of this kind becomes more difficult as the number of model parameters increases. Nevertheless, given the power available within the dolphin data-set, we believe that our implementation can often recover useful information when the number of model parameters is sufficiently low (i.e., when $d = 2$ and we are dealing with only pairs of variables).

To demonstrate the effectiveness of our implementation we perform a numerical experiment involving multiple trials. For each trial we fix $d = 2$ (corresponding to a pair of variables), set $\boldsymbol{a} \in \mathbb{R}^d$ to be the zero-vector, and select $A \in \mathbb{R}^{d\times d}$, $B \in \mathbb{R}^{d\times d}$ and $C \in \mathbb{R}^{d\times d}$ randomly. When randomly selecting A we constrain the eigenvalues of A to have negative-real-part (so that the resulting SDE produces bounded trajectories) and fix the frobenious norm of A to be 1. When randomly

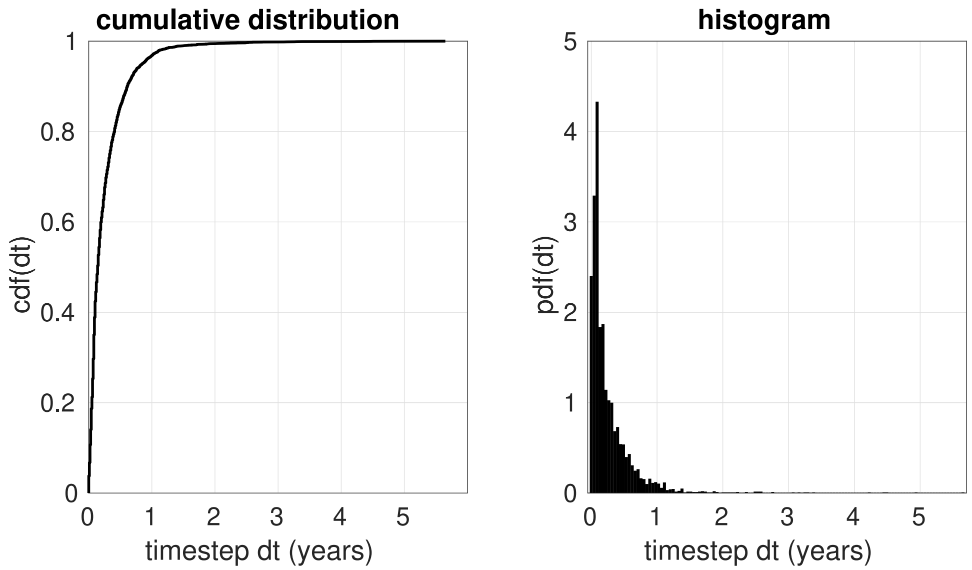

Fig A. Illustration of the distribution of time-steps within the dolphin data (aggregated across all dolphins). The cumulative-distribution-function is shown on the left and a histogram is shown on the right. This distribution is quite similar to an exponential-distribution with mean ~ 0.257 years. Additionally, for most of the dolphins with many measurements, only a small number (i.e., $\sim 2\%$) of the time-steps are identically zero (corresponding to non-unique observed-times τ_j).

selecting B and C we enforce symmetry, but do not constrain the magnitude of B and C (i.e., B and C can have frobenious norm less than or greater than 1). After selecting A, we generate a random trajectory $X(t)$ from the SDE shown in Eq 1 (i.e., using a randomly chosen realization of the weiner process $W(t)$). We then sample observed-times from this trajectory, using both the number and distribution of observed-times from the dolphin data-set. Thus, we sample ~ 5300 observed-times τ_j, corresponding to ~ 5200 distinct times t_k, with nonzero time-steps $\Delta t_k = t_{k+1} - t_k$ distributed roughly exponentially with a mean of 0.257 years (see Fig A). For each observed-time τ_j we use $X_{k(j)}$ and C to sample Y_j as shown in Eq 2. We then remove roughly $\sim 2\%$ of the observed measurements at random (i.e., components of the various Y_j), treating these as missing (in accordance with the dolphin data-set).

From the data $\{\tau_j\}$ and $\{Y_j\}$ we then use our methods described above to estimate the model parameters α, A, B and C (with $q_{\max} = 0$). Once we have estimated the model parameters, we compare the estimated results to the true parameter values used for that trial. For each trial we measure the correlation $\rho(A)$ between the estimated- and true-values for A. We also measure the ratio $\sigma(A; [B, C])$ between the frobenius-norm of the true value of A and the frobenius-norm of the true value of $[B, C]$:

$$\sigma(A; [B, C]) = \frac{\sqrt{\|A\|_F^2}}{\sqrt{\|B\|_F^2 + \|C\|_F^2}}.$$

For any particular trial the value of $\sigma(A; [B, C])$ can be thought of as a version of a 'signal-to-noise ratio', while the value of $\rho(A)$ measures the recovery quality for A.

An example of these results (aggregated over 25600 random trials) is shown in Fig B. For this figure we first bin the trials by their signal-to-noise $\sigma(A; [B, C])$, shown along the horizontal. For each of these bins we construct a histogram with respect to $\rho(A)$. Each column of the heatmap in Fig B shows one of these histograms; the color indicates log2-density (see colorbar on the right). The median of each histogram is indicated in thick cyan, with the 85%-ile and 15%-ile shown in thin

cyan. Note that when A is roughly the same size as $[B, C]$ (i.e., when snr ~ 1, or $-\log_{10}(\text{snr}) \sim +0.0$) then the recovery is quite good (i.e., close to 100%). When A is only one-tenth the size of $[B, C]$ (i.e., when snr $\sim 1/10$, or $-\log_{10}(\text{snr}) \sim +1.0$) the typical correlation drops to $\sim 85\%$ or so. When A is only one-hundredth the size of $[B, C]$ (i.e., when snr $\sim 1/100$, or $-\log_{10}(\text{snr}) \sim +2.0$) the recovery is quite poor, and many trials have a correlation of less than 50%.

We represent the same numerical experiments in a different format within Fig C. In this figure we first divide the trials into three categories. The first category (i.e., 'B small') corresponds to B less than twice the size of A (i.e,. $\|B\|_F \leq 2$). The second category (i.e., 'B medium') corresponds to B between two and eight times the size of A (i.e,. $2 \leq \|B\|_F \leq 8$). The third category (i.e., 'B large') corresponds to B more than eight times the size of A (i.e,. $8 \leq \|B\|_F$). These three categories are shown in the left, middle and right subplots, respectively. For each category we measure the frobenius-norm $\|C\|_F$, which can be thought of as the inverse of the signal-to-noise ratio relating A to C (recall that $\|A\|_F$ was fixed at 1). For each value of $\|C\|_F$ (shown along the horizontal) we again construct a histogram with respect to $\rho(A)$. Once again, each column showns the log2-density of the associated histogram of $\rho(A)$ for that value of $\|C\|_F$, with the 15, 50 and 85 percentiles indicated in cyan. Note that the recovery of A is typically quite good when B and C are each only a few times larger than A. However, when either B or C is very large, then the recovery of A suffers.

In the case of the dolphin data-set, we believe that B and C are typically between 1 and 6 times bigger than A, meaning that $\sigma(A, [B, C]) \lesssim 10$, with values of $\|C\|_F / \|A\|_F \lesssim 0.5$. Given the power of the dolphin data-set (i.e., the number of observed-times), this range of model parameters corresponds to typical values of $\rho(A)$ around 80% or so (see Figs B and C). Thus, we feel reasonably safe reporting our results when $d = 2$.

By contrast, our methods are not sufficiently sensitive to accurately recover the parameters for models involving more than two variables simultaneously. As an example, we repeat the numerical experiments above for the case with $d = 3$ (i.e., three interacting variables). The results are shown in Fig D. Given the power available within the dolphin data-set, we do not typically achieve high recovery quality, even when B and C are not much bigger than A.

4 Biclustering the results

In this section we describe the methods we use to identify 'push-pull' blocks within the array of type-A interactions. As an example of the structures we are trying to identify, see Fig E.

Our overall strategy is adapted from the 'loop-counting' strategy described in [3], giving rise to an unsupervised 'top-down' method, and a supervised (or user-informed) 'bottom-up' method. The essential idea is to (i) develop a 'score' for each row of the array which is correlated with the likelihood that that particular row participates in the push-pull block, and (ii) do the same for the columns.

The top-down method involves calculating these scores across the entire array, then pruning the array by iteratively eliminating rows and columns with the lowest scores. By contrast, the bottom-up method works in the reverse direction, starting with a small collection of user-provided rows and columns (i.e., an initial estimate or 'seed' for the push-pull block) and then growing the push-pull block by iteratively adding the rows and columns with the highest scores.

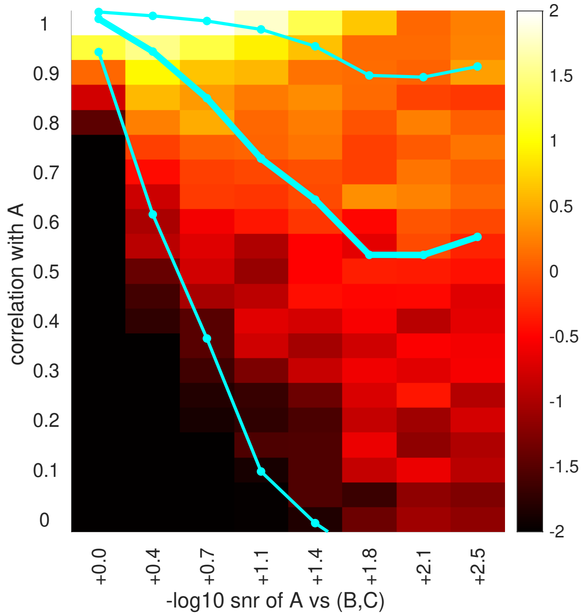

Fig B. Here we summarize the results of the numerical experiments described in section 3.3 for $d = 2$. The horizontal axis shows a signal-to-noise (snr) comparing the magnitude of the true A to that of the true B and C. For each horizontal location we show a vertical column indicating the histogram of correlations betweeen the estimated and true A. The heatmap corresponds to the log2-density of these histograms. The cyan lines indicate the 15, 50 and 85 percentiles for these histograms. Note that when A is roughly the same size as $[B, C]$ then the recovery is quite good (i.e., close to 100%). However, if A is many times smaller than $[B, C]$ then the recovery suffers.

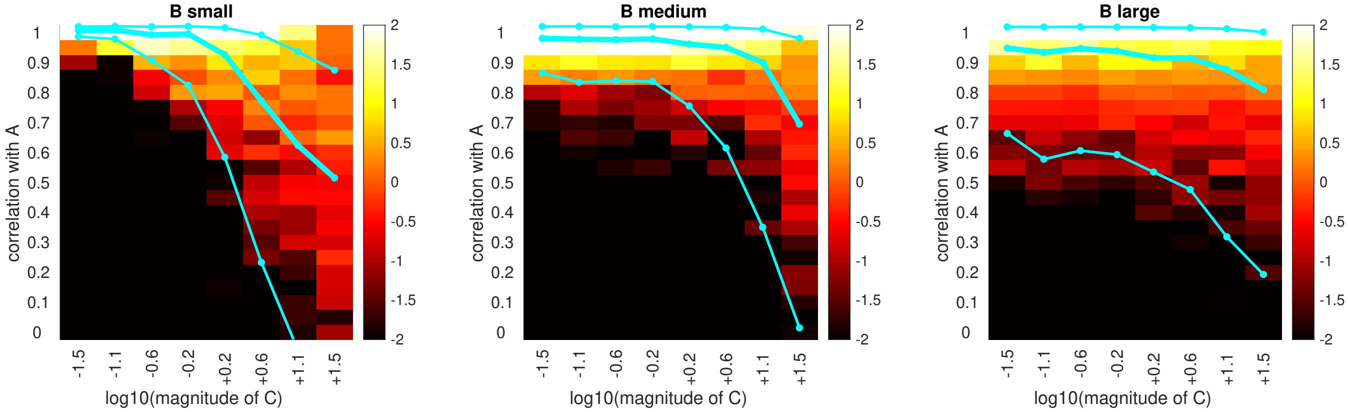

Fig C. Here we show a different representation of the same numerical experiment from Fig B, involving $d = 2$. This time we divide the trials into three categories, correspond to B small, medium and large (left, center and right subplots, respectively). For each category we sort the trials in that category by the frobenius-norm of C (shown along the horizontal). For each value of $\|C\|_F$ we construct a histogram of the recovery quality (vertical). The 15, 50 and 85 percentiles of these histograms are shown in cyan. Note that the recovery of A is typically quite good, except when either B or C is much larger than A.

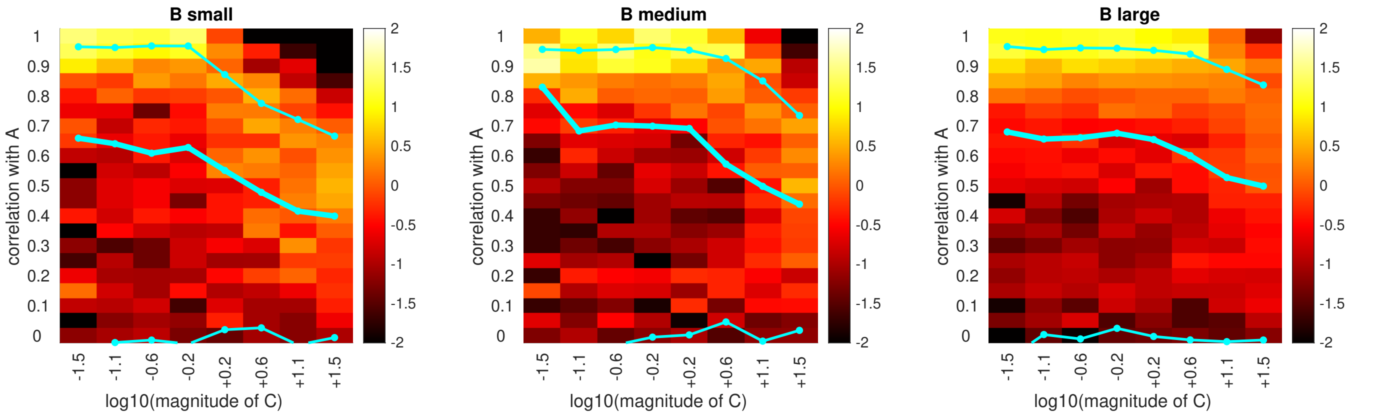

Fig D. Here we show results for numerical experiments with $d = 3$. The format for this figure is analogous to Fig C. Note that the recovery of A is not particularly good, even when both B and C are of moderate size.

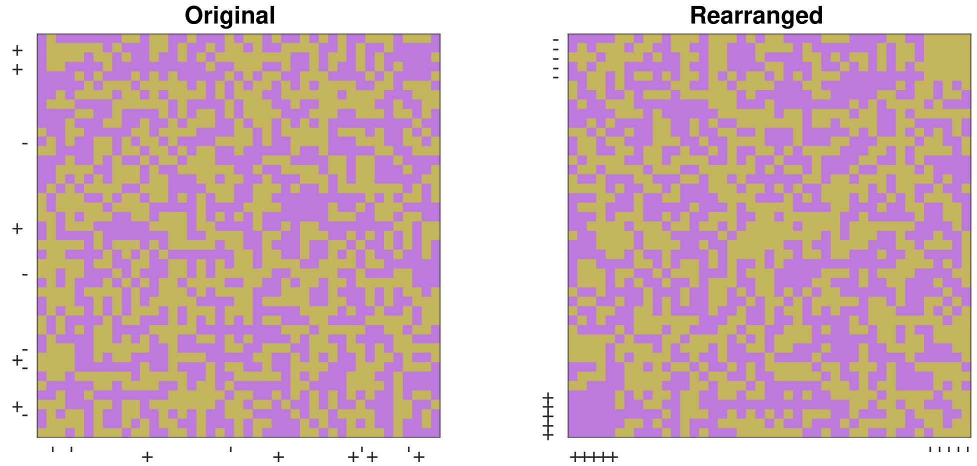

Fig E. On the right we show a simulated array of type-A interactions. This array is generated by first taking a random matrix (with each entry drawn independently), and then planting a small push-pull block. To define this push-pull block, we first randomly select 2 subsets of 5 variables each, denoted '$\mathcal{V}$' and '$\mathcal{V}'$', respectively. Once we have defined $\mathcal{V}$ and $\mathcal{V}'$, we fix the interactions between any source variable from set $\mathcal{V}$ and any target variable from set $\mathcal{V}'$ to be excitatory, while fixing the reciprocal interaction to be inhibitory. The variables corresponding to $\mathcal{V}$ and $\mathcal{V}'$ are indicated with the '+' and '-' tick-marks along the axes. If these variables can be identified, then the original array can be re-organized to reveal $\mathcal{V}$ and $\mathcal{V}'$ as a pair of contiguous submatrices (see left subplot). In this particular example the original size of the array is $N \times N$, with $N = 43$, similar to the number of variables used in the dolphin data. The number of interactions within the planted push-pull block is roughly N^M, with $M = 0.5$, corresponding to the detection-threshold of our top-down algorithm in the large N limit (see [3] and Fig F). We expect our top-down algorithm to reliably find push-pull blocks that are bigger than this threshold when N is sufficiently large. We expect our bottom-up algorithm to reliably complete push-pull blocks of this size for a wide range of N, assuming that the initial estimate for the push-pull block is a sufficiently large subset of the full block.

In terms of details, we will aim to find sets $\mathcal{V}$ and $\mathcal{V}'$ such that the quality Z_{all}^2 is large, with Z_{all} given by:

$$Z_{\text{all}}(\mathcal{V},\mathcal{V}') = \sum_{v\in\mathcal{V}}\sum_{v'\in\mathcal{V}'}\left(A_{vv'} - A_{v'v}\right). \quad (16)$$

The quality Z_{all}^2 will be large when $\mathcal{V}$ and $\mathcal{V}'$ form a push-pull block, with interactions $A_{vv'}$ typically of opposite sign to their reciprocal interactions $A_{v'v}$.

To search for push-pull blocks, we'll separate this measure of quality into row-scores:

$$Z(v;\mathcal{V},\mathcal{V}') = \sum_{v'\in\mathcal{V}'}\left(A_{vv'} - A_{v'v}\right), \quad (17)$$

and column-scores:

$$Z'(v';\mathcal{V},\mathcal{V}') = \sum_{v\in\mathcal{V}}\left(A_{vv'} - A_{v'v}\right). \quad (18)$$

With these definitions one can immediately see that:

$$Z_{\text{all}} = \sum_{v\in\mathcal{V}} Z(v) = \sum_{v'\in\mathcal{V}'} Z'(v').$$

4.1 Top down:

These definitions motive a very simple 'top-down' method for finding push-pull blocks:

Initialize: Define both $\mathcal{V}$ and $\mathcal{V}'$ to be the entire set of variables, and set the iteration $\mu = 0$.

Calculate scores: Use Eq 17 to define row-scores for each variable in $\mathcal{V}$ and use Eq 18 to define column-scores for each variable in $\mathcal{V}'$. Along the way record the quality $Z_{\text{all}}^2(\mu)$ for the current iteration μ (i.e., for the current sets $\mathcal{V}$ and $\mathcal{V}'$).

Eliminate the lowest scoring variable: Select one of the elements in either $\mathcal{V}$ or $\mathcal{V}'$ with the lowest score, and eliminate it.

Iterate: Iterate this process, recalculating the scores and eliminating the variable (from either $\mathcal{V}$ or $\mathcal{V}'$) corresponding to the lowest score.

This process will result in a list of variables in the order they were eliminated.

Under certain assumptions, the variables that are retained the longest by the top-down algorithm will form a push-pull block. Because of the similarities between this strategy and the loop-counting methods described in [3], many of the same analytical arguments can be slightly modified to apply to this scenario. For example, the sensitivity of this algorithm is quite similar to spectral clustering [4], and we can make the following statistical claim: If we are given a large random array with a sufficiently large push-pull block hidden within it, then this algorithm will often retain the variables within the push-pull block, eliminating the other variables first. In the limit as the number of variables N goes to infinity, this

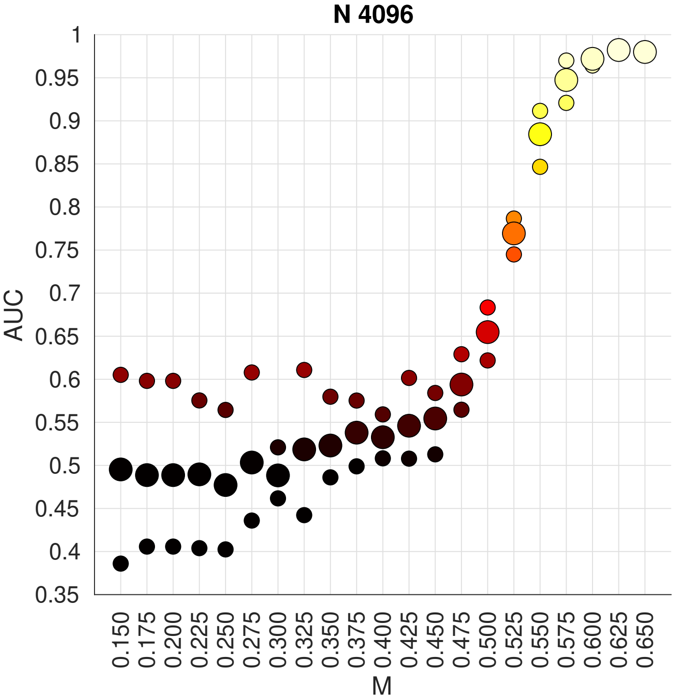

Fig F. In this figure we illustrate the sensitivity of our top-down loop-counting algorithm for detecting push-pull blocks (see text). For each value of M we collect multiple random trials (with different samples of A, $\mathcal{V}$ and $\mathcal{V}'$) and run our top-down algorithm, measuring the average AUC. The median value is shown with large dots, while the 15%-ile and 85%-ile are shown in smaller dots above and below. Note that the detection-threshold for our algorithm is $M \sim 0.5$, corresponding to push-pull blocks with roughly $\sqrt{N/2}$ variables within each of them.

algorithm will succeed with a probability exponentially close to 1 when the number of variables in the push-pull block is $\gtrsim \sqrt{N/2}$ (i.e., when the number of push-pull interactions in the block is $\gtrsim N$).

To demonstrate this detection-threshold we conduct a numerical experiment. For each trial of this numerical experiment we randomly generate a binary array A of size $N \times N$, with each entry chosen independently from $[-1, +1]$. Within this array we implant a push-pull block with size $n = |\mathcal{V}| = |\mathcal{V}'|$ determined by the parameter $M \in [0, 1]$. For a particular value of M, we set $n = \lceil N^M/\sqrt{2} \rceil$, such that $2n^2 \approx N^{2M}$. Thus, the number of push-pull interactions in the planted block is approximately equal to the Mth power of the total number of interactions in the original array.

Once we have a random binary matrix A with a planted block, we run the algorithm described above. We record the list of variables as they are eliminated. After recording this list, we measure the AUC between (i) the listed rank of the variables *not* in $\mathcal{V}$, and (ii) the listed rank of the variables *in* $\mathcal{V}$. We then measure the AUC' similarly for $\mathcal{V}'$, and take the average of AUC and AUC'. If this average AUC is 1, then our algorithm was perfectly successful (i.e., all the variables within the planted block were retained until the very end). If this average AUC is 0.5, then our algorithm performed no better than chance (i.e., the variables within the planted block were not retained any longer than the other variables were).

Results of this numerical experiment are shown in Fig F. This figure plots the average AUC mentioned above as a function of M, for $N = 4096$. Note that as M approaches 0.5 (and n approaches $\sqrt{N/2}$), the recovery of our algorithm increases.

In practice, we can determine which variables might form a push-pull block by using a permutation-test. For this permutation-test we compare the quality $Z^2_{\text{all}}(\mu)$ to the distribution of $Z^2_{\text{all}}(\mu)$ observed when running the algorithm on

randomly reorganized versions of the original matrix. The iteration μ for which the original quality $Z^2_{\text{all}}(\mu)$ is most significant (relative to the distribution of $Z^2_{\text{all}}(\mu)$ from the randomly reorganized samples) is a natural candidate for the μ one should use to identify the entries in the push-pull block. See, e.g., Figs 10 and 11 and the Supplementary Information in [3] for a more detailed description.

4.2 Bottom up:

In addition to the top-down algorithm described above, we can also use the row- and column-scores to construct a 'bottom-up' algorithm using a strategy similar to many modularity-maximization algorithms (such as louvain clustering [5]):

Initialize: Define $\mathcal{V}$ and $\mathcal{V}'$ to each be a user-specified group of variables, and set the iteration $\mu = 0$.

Calculate scores: Use Eq 17 and 18 to define row- and column-scores for each variable in the full set of variables. Along the way record the quality $Z^2_{\text{all}}(\mu)$ for the current iteration μ (i.e., for the current sets $\mathcal{V}$ and $\mathcal{V}'$).

Add the highest scoring variable: Select one of the values of $Z(v)$ or $Z'(v')$ with the highest score, and add the corresponding variable to $\mathcal{V}$ or $\mathcal{V}'$, respectively.

Iterate: Iterate this process, recalculating the scores and adding variables (to either $\mathcal{V}$ or $\mathcal{V}'$) corresponding to the highest score.

This process will result in a list of variables in the order they were added.

Under certain assumptions, the variables that are added first by the bottom-up algorithm will form a push-pull block. For example, if (i) we are given a large random array with a push-pull block hidden within it, and (ii) the inital push-pull block is a sufficiently large subset of the planted block, then (iii) this algorithm will add the variables within the push-pull block first, ignoring the other variables until later. In the limit as the number of variables N goes to infinity, this algorithm will succeed with a probability exponentially close to 1 when (i) the initial block is a subset of the planted block, and (ii) the number of variables in the initial block is $\gtrsim \mathcal{O}(\log_2 N)$.

In practice, we can once again use a permutation-test to determine which variables are within the push-pull block. For this permutation-test we compare the quality $Z^2_{\text{all}}(\mu)$ to the distribution of $Z^2_{\text{all}}(\mu)$ observed when running the algorithm on randomly reorganized versions of the original matrix (where all the entries other than those in the initial block are permuted).

4.3 Analyzing the type-A interactions

We can use the strategies described above to conduct an exploratory analysis of the push-pull blocks within the type-A interactions from the main manuscript. To do so, we first run the top-down algorithm from section 4.1, identifying those variables retained the longest and grouping them into a putative push-pull block. Then we run the bottom-up algorithm from section 4.2 multiple times, using as initial seeds each pair of variables within the putative push-pull block identified earlier. We also run the bottom-up algorithm using as initial seeds each pair of variables that could form a push-pull block themselves (similar in spirit to market basket analysis [6]).

After performing this analysis we recover a large number of slightly different push-pull blocks. Each of these blocks is statistically significant in its own right, but they are far from distinct (indeed, many of these push-pull blocks overlap strongly with one another). While these different push-pull blocks can certainly be merged (using, e.g., the criteria from [7,8]), we find that the final results of merging push-pull blocks is not robust (i.e., the identity of the final push-pull blocks can change substantially if the merging criteria are altered slightly).

To dodge this issue, we simply step through the list of significant push-pull-blocks, grabbing the largest remaining push-pull block and removing it until nothing of significance remains. This process allows us to order the variables from the original data-set so that several of the most significant push-pull blocks are visually obvious. One such arrangement is shown in Fig 8 in the main text.

When assessing the array of type-A interactions, we can summarize the significance of any particular push-pull block independently, without relying on the methodology used to detect that push-pull block. Given a push-pull block, we define the p-value p_r to be the probability that a push-pull block of at least the same size exists within a random array with entries drawn independently (with replacement) from the original A-array. An upper-bound (i.e., conservative estimate) for p_r, denoted p_u, can be constructed using a simple union bound.

To constuct p_u we introduce the following notation:

1. The original $N \times N$ array of type-A interactions has a fraction f_+ of positive entries, and a fraction f_- of negative entries. Note that f_+ and f_- need not necessarily add up to 1.

2. The push-pull block has sizes $|\mathcal{V}|$ and $|\mathcal{V}'|$ denoted by V and V', respectively.

3. The block of interactions $\{A_{v'v}\}$ between source-variables from $\mathcal{V}$ and target-variables from $\mathcal{V}'$ has n_+ positive entries.

4. The block of interactions $\{A_{vv'}\}$ between source-variables from $\mathcal{V}'$ and target-variables from $\mathcal{V}$ has n_- negative entries.

With these assumptions we can bound p_r via:

$$p_r \leq p_u = \frac{N!}{(N-V-V')! \cdot V! \cdot V'!} \left[\sum_{n=n_+}^{n=VV'} \binom{VV'}{n} \cdot f_+^n \cdot (1-f_+)^{VV'-n} \right] \left[\sum_{n=n_-}^{n=VV'} \binom{VV'}{n} \cdot f_-^n \cdot (1-f_-)^{VV'-n} \right] \quad (19)$$

As an example of this upper-bound, we can consider the holm-bonferroni corrected array of type-A interactions for which f_+ and f_- are each less than 0.10. The push-pull cluster defined by $\mathcal{V}$ containing RBC, HGB and HCT, and $\mathcal{V}'$ containing AST, MCH, Bilirubin, ALT, Sed60 and Iron has $V = 3$, $V' = 6$, $n_+ = 18$ and $n_- = 16$; the corresponding upper bound $p_u < \exp(-50) \sim 2 \times 10^{-22}$. The push-pull cluster defined by $\mathcal{V}$ containing AlkPhos and InorgPhos, and $\mathcal{V}'$ containing CPK, Platelets and BUN has $V = 2$, $V' = 3$, $n_+ = 6$ and $n_- = 4$; the corresponding upper bound $p_u = 0.007$. The push-pull cluster defined by $\mathcal{V}$ containing Creatinine and Lymphs, and $\mathcal{V}'$ containing AlkPhos, InorgPhos, CPK and Platelets has $V = 2$, $V' = 4$, $n_+ = 6$ and $n_- = 6$; the corresponding upper bound $p_u = 0.026$. Note that not all visually identifiable push-pull clusters have a low upper-bound p_u. For example, the push-pull cluster defined by $\mathcal{V}$ containing

MCV, Monocytes and ACMonocytes, and $\mathcal{V}'$ containing RBCDist, Mg and Potassium has $V = 3$, $V' = 3$, $n_+ = 8$ and $n_- = 3$, yet the corresponding upper bound $p_u = 0.28$.

5 Holm-Bonferroni Adjustment

As mentioned in the main text, the parameters observed for any label-shuffled trial are not uncorrelated with one another. Consequently, the standard bonferroni-corrected p-value p_b is an overestimate (i.e., p_b is too conservative). We calculate a more accurate adjusted p-value 'p_h' by using an empirical version of the holm-bonferroni adjustment.

To describe this in detail consider the collection of $J = N(N-1)$ parameters $A_{vv'}$ for all variable-pairs $v \neq v'$. We first determine the J-element vector of these parameters for the original-data (by fitting each variable-pair individually). We'll refer to this vector of parameters as $\mathcal{A}_{:,0}$, with $\mathcal{A}_{j,0}$ referring to the jth parameter from the original data, and the colon ':' referring to 'all rows'. We then determine the corresponding vector for each of the K label-shuffled trials. We'll refer to each of these vectors as $\mathcal{A}_{:,k}$ for $k \in 1, \ldots, K$, one for each trial. Together, the $\mathcal{A}_{:,k}$ form a $J \times K$ array $\mathcal{A}_{:,:}$, with $\mathcal{A}_{j,k}$ referring to the jth parameter from trial k (the second colon on $\mathcal{A}_{:,:}$ refers to 'all columns').

To put these J different parameters on equal footing we first convert them all to z-scores; for each j we convert all the entries of $\mathcal{A}_{j,0}$ and $\mathcal{A}_{j,:}$ into z-scores using the Gaussian-distribution fit to the collection of K entries in the row-vector $\mathcal{A}_{j,:}$. After converting each entry to a z-score, we apply a 2-sided test to convert each z-score to a negative-log-p-value $l = \text{erfcln}(|z|/\sqrt{2})$. This procedure produces a vector of negative-log-p-values $\boldsymbol{l}_{j,0}$ corresponding to the original data, as well as an array $\boldsymbol{l}_{j,:}$ corresponding to the label-shuffled trials. Higher values of l indicate more significant values.

Now we sort $\boldsymbol{l}_{:,0}$, as well as each of the $\boldsymbol{l}_{:,k}$ in the j-direction, in descending order. We'll refer to these j-sorted vectors as $\boldsymbol{l}'_{:,0}$ and $\boldsymbol{l}'_{:,k}$.

For each rank-j (ranging from the largest and most significant at rank-1 to the smallest and least significant at rank-J), we fit the K sorted negative-log-p-values $\boldsymbol{l}'_{j,:}$ with a gumbel-distribution, denoted by $G_j(l')$. We then use the distribution $G_j(\cdot)$ to ascribe a (one-sided) p-value p'_h to the value $\boldsymbol{l}'_{j,0}$. Finally, we set the adjusted p-value p_h to be $p_h = \max(p'_h, p_0)$.

We can confirm that our holm-bonferroni adjusted p-value p_h is more accurate than the original bonferroni-adjusted p_b by measuring the fraction $P_h(x)$ of label-shuffled trials with at least one holm-bonferroni adjusted p-value (taken across the J variable-pairs) less than x. This cumulative-distribution function is quite close to the line $P_h(x) = x$, indicating that p_h is an accurate estimate of the true p-value, after adjusting for multiple hypotheses (e.g., see Fig G).

5.1 Estimating Significant Differences

To search for significant differences between two different subsets of dolphins, we employ the same strategy described above, except applied to the difference between parameters (rather than the parameters themselves). Additionally, when comparing two different subsets we use all pairs of permutations π_k,$\pi_{k'}$ to define the label-shuffled distribution.

To be more explicit, imagine that the first set is 'dolphins between the ages of 10 and 30', while the second set is 'dolphins over the age of 30'. We'll denote these two sets by S^1 and S^2. Continuing with the example we used above when discussing the holm-bonferroni adjustment, we would measure the J parameters $A_{vv'}$ (across all variable-pairs v, v') for

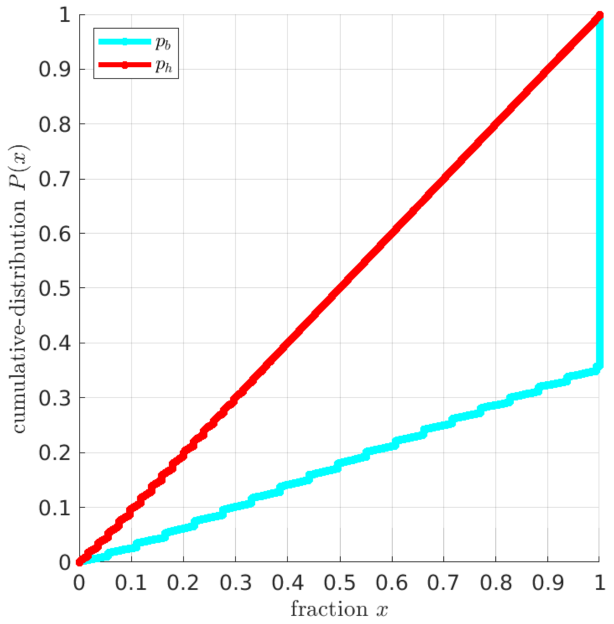

Fig G. Here we illustrate (in red) the cumulative-distribution $P_h(x)$ for the holm-bonferroni adjustment, as calculated for the deterministic interaction terms $A_{vv'}$ across all variable-pairs. This cumulative-distribution $P_h(x)$ is defined to be the fraction of label-shuffled trials exhibiting at least one value of p_h (considered across all variable-pairs) less than x. The p-values refer to the significance of the difference (in parameters) between the subsets S^2 and S^1. The subset $\mathcal{S}^2$ refers to all dolphins over the age of 30, while the subset $\mathcal{S}^1$ refers to dolphins between the ages of 10 and 30. An analogous cumulative-distribution $P_b(x)$ for the bonferroni-correction is shown in cyan. Note that the value of $P_h(x)$ closely aligns with the identity line (grey).

each set, denoting the results with the vectors $\mathcal{A}^1_{:,0}$ and $\mathcal{A}^2_{:,0}$, respectively (note that here the superscript does not indicate an exponent, but rather the subset considered). We also do the same for each of the K label-shuffled trials, producing the arrays $\mathcal{A}^1_{:,:}$ and $\mathcal{A}^2_{:,:}$. Before proceeding any further we calculate the differences $\delta\mathcal{A}_{:,0} = \mathcal{A}^2_{:,0} - \mathcal{A}^1_{:,0}$ for the original data, and the K^2 differences $\delta\mathcal{A}_{:,k''} = \mathcal{A}^2_{:,k} - \mathcal{A}^1_{:,k'}$ for each pair of label-shuffled trials, where the index $k'' = 1, \ldots, K^2$ enumerates all the trial-pairs k, k'.

From here on we proceed as usual, replacing (respectively) the vector $\mathcal{A}_{:,0}$ and the $J \times K$ array $\mathcal{A}_{:,:}$ with the vector $\delta\mathcal{A}_{:,0}$ and the $J \times K^2$ array $\delta\mathcal{A}_{:,:}$. An example of adjusted p-values produced using this approach is shown in Fig G.

6 Estimating Aging-rate

To demonstrate consistency with the analysis of [9], we measure the slope of the age-related drift for the 6 biomarkers Hemoglobin (HGB), Alkaline Phosphatase, Platelets, Lymphocytes, Creatinine and Protein across each of the dolphins from age 10yr onwards. We project all dolphins onto the first principal-component 'u' of this array, separating them into two categories based on the median of u. Those dolphins with larger-than-median u-values typically exhibit slow deterioration of Hemoglobin, Alkaline Phosphatase, Platelets and Lymphocytes, along with a slow accumulation of Creatinine and Protein; these are classified as slow-agers. Conversely, the dolphins with lower-than-median u-values typically exhibit rapid deterioration of Hemoglobin, Alkaline Phosphatase, Platelets and Lymphocytes, along with rapid accumulation of Creatinine and Protein; these are classified as accelerated-agers. As shown in Fig H, the accelerated-agers tend to develop

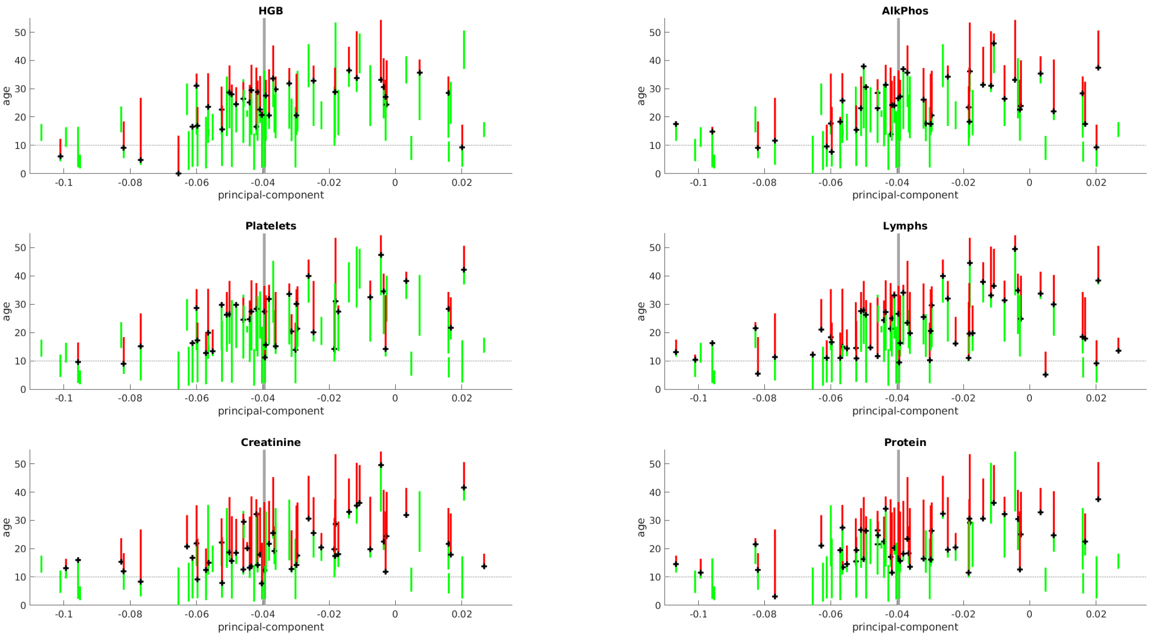

Fig H. Here we illustrate the distribution of dolphins with regards to aging rate. As described in the Methods, we have measured the slope of the age-related drift for the 6 biomarkers HGB, AlkPhos, Platelets, Lymphocytes, Creatinine and Protein, using only measured ages above 10 years. We calculated the first principal-component u of this array, and projected each dolphin onto this principal-component. In each subplot we illustrate the correlation between age-related conditions and the u-value for each dolphin. Taking the first subplot (HGB) as an example, each dolphin is illustrated using a vertical line positioned at that dolphin's u-value. Each vertical line spans the dolphin's measured ages, with green segments indicating ages where the dolphin exhibits normal values of HGB, and red segments indicating ages after which the dolphin first exhibited a low value of HGB (i.e., anemia). The threshold we use to distinguish normal HGB from anemia is the value of HGB=12 listed in table-2 of [9]. For visual clarity we place a black '+' at the age when each dolphin first exhibits a low HGB value. One can clearly see a correlation between the u-value for each dolphin and the age at which that dolphin first exhibits anemia. The remaining subplots are analogous to the first, referencing the other 5 age-related variables described in table-2 of [9]. In the background of each subplot we highlight the median value of $u \sim -0.04$, which we use as a threshold to categorize dolphins into slow-agers ($u > -0.04$) and accelerated-agers ($u < -0.04$).

anemia and lymphopenia and clinically low levels of alkaline phosphatase and platelets more rapidly than the slow-agers.

References

1. Gardiner CW. Handbook of stochastic methods for physics, chemistry and the natural sciences. vol. 13 of Springer Series in Synergetics. 3rd ed. Berlin: Springer-Verlag; 2004.

2. Dempster AP, Laird NM, Rubin DB. Maximum Likelihood from Incomplete Data Via the EM Algorithm. Journal of the Royal Statistical Society: Series B (Methodological). 1977;39(1):1–22. doi:https://doi.org/10.1111/j.2517-6161.1977.tb01600.x.

3. Rangan AV, McGrouther CC, Kelsoe J, Schork N, Stahl E, Zhu Q, et al. A loop-counting method for covariate-corrected low-rank biclustering of gene-expression and genome-wide association study data. PLOS Computational Biology. 2018;14(5):1–29. doi:10.1371/journal.pcbi.1006105.

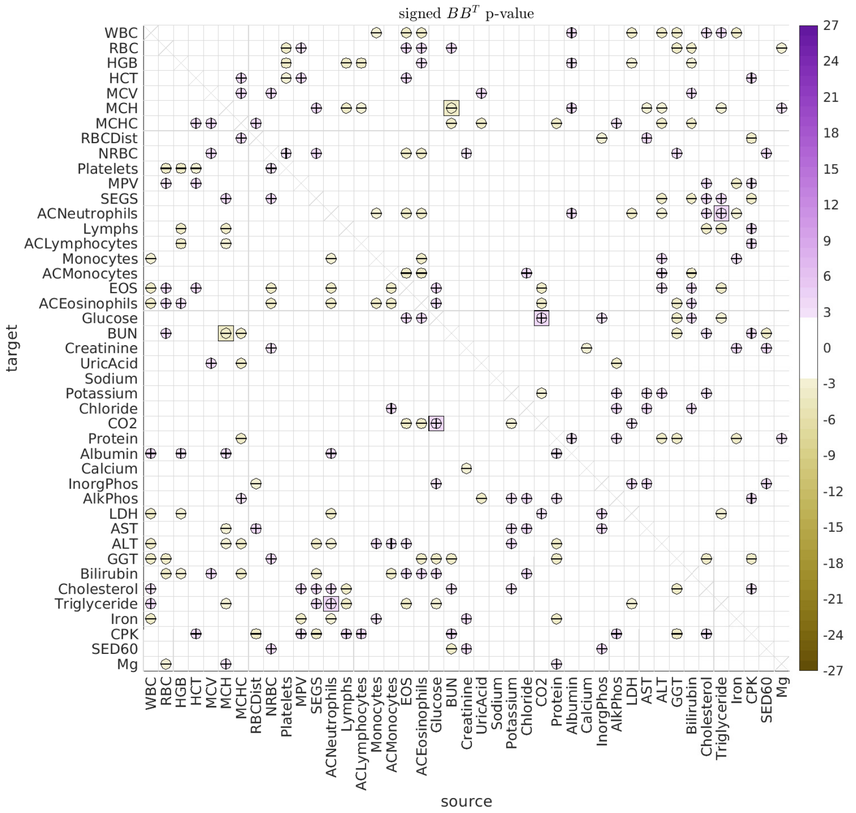

Fig I. This figure has the same format as Fig 7 in the main text, showing the significant differences in the stochastic correlations $[BB^{\intercal}]_{vv'}$ between (i) dolphins over age 30 and (ii) dolphins between the ages of 10 and 30.

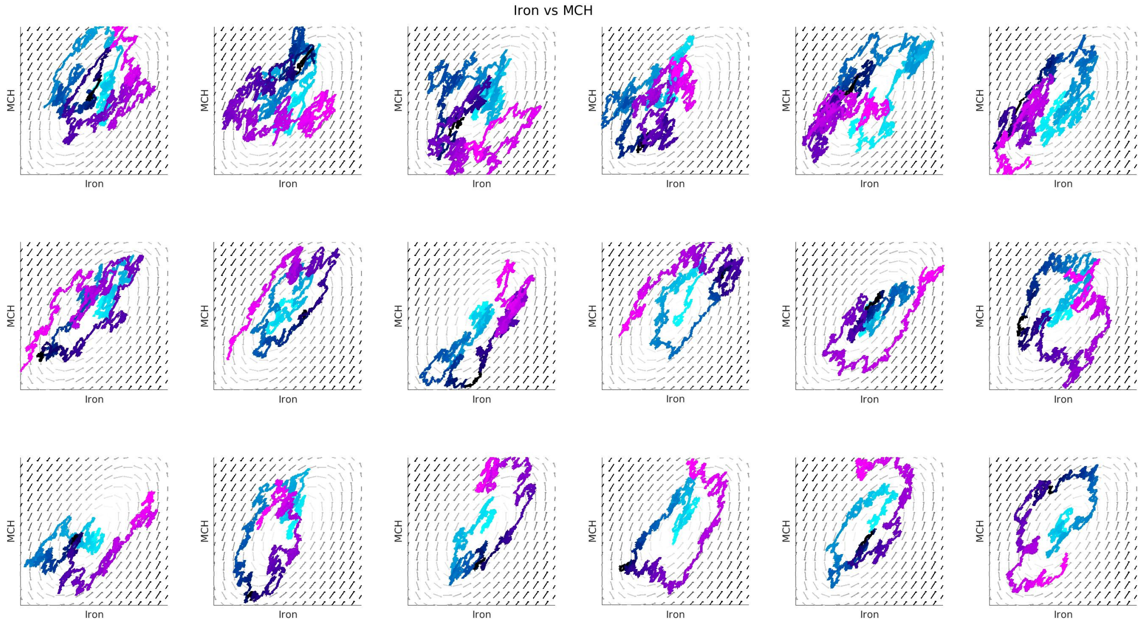

Fig J. In this figure we show many different realizations of the stochastic differential equation (SDE) relating Iron to MCH. Each panel in this figure has the same format as the left side of Fig 4 from the main text.

4. Alon N, Krivelevich M, Sudakov B. Finding a large hidden clique in a random graph. Random Structures & Algorithms. 1998;13(3-4):457–466. doi:https://doi.org/10.1002/(SICI)1098-2418(199810/12)13:3/4¡457::AID-RSA14¿3.0.CO;2-W.

5. Blondel VD, Guillaume JL, Lambiotte R, Lefebvre E. Fast unfolding of communities in large networks. Journal of Statistical Mechanics: Theory and Experiment. 2008;2008(10):P10008. doi:10.1088/1742-5468/2008/10/p10008.

6. Larose DT, Larose CD. Discovering Knowledge in Data: An Introduction to Data Mining, Second Edition. John Wiley & Sons, Inc.; 2014.

7. Magland JF, Barnett AH. Unimodal clustering using isotonic regression: ISO-SPLIT. technical report. 2016;.

8. Chung JE, Magland JF, Barnett AH, Tolosa VM, Tooker AC, Lee KY, et al. A fully automated approach to spike sorting. Neuron. 2017;95:1381–1394.

9. Venn-Watson S, Jensen ED, Schork NJ. A 25-y longitudinal dolphin cohort supports that long-lived individuals in same environment exhibit variation in aging rates. Proceedings of the National Academy of Sciences. 2020;117(34):20950–20958. doi:10.1073/pnas.1918755117.
